# Cupping in the Monkey Optic Nerve Transection Model Consists of Prelaminar Tissue Thinning in the Absence of Posterior Laminar Deformation

**DOI:** 10.1167/iovs.15-18975

**Published:** 2016-05-11

**Authors:** Eliesa Ing, Kevin M. Ivers, Hongli Yang, Stuart K. Gardiner, Juan Reynaud, Grant Cull, Lin Wang, Claude F. Burgoyne

**Affiliations:** 1 Discoveries in Sight Research Laboratories, Devers Eye Institute, Legacy Research Institute, Portland, Oregon, United States; 2 Optic Nerve Head Research Laboratory, Devers Eye Institute, Legacy Research Institute, Portland, Oregon, United States

**Keywords:** glaucoma, lamina cribrosa, optical coherence tomography, optic nerve transection, cupping

## Abstract

**Purpose:**

To use optical coherence tomography (OCT) to test the hypothesis that optic nerve head (ONH) “cupping” in the monkey optic nerve transection (ONT) model does not include posterior laminar deformation.

**Methods:**

Five monkeys (aged 5.5–7.8 years) underwent ONH and retinal nerve fiber layer (RNFL) OCT imaging five times at baseline and biweekly following unilateral ONT until euthanization at ∼40% RNFL loss. Retinal nerve fiber layer thickness (RNFLT) and minimum rim width (MRW) were calculated from each pre- and post-ONT imaging session. The anterior lamina cribrosa surface (ALCS) was delineated within baseline and pre-euthanasia data sets. Significant ONT versus control eye pre-euthanasia change in prelaminar tissue thickness (PLTT), MRW, RNFLT, and ALCS depth (ALCSD) was determined using a linear mixed-effects model. Eye-specific change in each parameter exceeded the 95% confidence interval constructed from baseline measurements.

**Results:**

Animals were euthanized 49 to 51 days post ONT. Overall ONT eye change from baseline was significant for MRW (−26.2%, *P* = 0.0011), RNFLT (−43.8%, *P* < 0.0001), PLTT (−23.8%, *P* = 0.0013), and ALCSD (−20.8%, *P* = 0.033). All five ONT eyes demonstrated significant eye-specific decreases in MRW (−23.7% to −31.8%) and RNFLT (−39.6% to −49.7%). Four ONT eyes showed significant PLTT thinning (−23.0% to −28.2%). The ALCS was anteriorly displaced in three of the ONT eyes (−25.7% to −39.2%). No ONT eye demonstrated posterior laminar displacement.

**Conclusions:**

Seven weeks following surgical ONT in the monkey eye, ONH cupping involves prelaminar and rim tissue thinning without posterior deformation of the lamina cribrosa.

We have previously proposed that the clinical phenomenon of “permanent” or “nonreversible” cupping, regardless of etiology, has two principal pathophysiologic components: prelaminar tissue thinning and laminar deformation and/or remodeling^1–3^ (Fig. 1). We define prelaminar tissue thinning to be that portion of cup enlargement which results from thinning of the prelaminar tissues due to physical compression and/or loss of retinal ganglion cell (RGC) axons. We define laminar deformation and remodeling to be that portion of cup enlargement which results from permanent laminar deformation and/or laminar insertion migration following connective tissue damage and/or remodeling.^2–6^ We have further proposed that these laminar alterations define a glaucomatous optic neuropathy and occur in a pattern that is governed by the distribution of IOP-related connective tissue stress and strain, regardless of the mechanism of initial connective tissue insult or the level of IOP at which that insult occurs.^2,7^ In this context, “glaucomatous cupping” is the term clinicians use to describe the clinical appearance and behavior the optic nerve head (ONH) assumes as its neural and connective tissues are deformed and/or remodeled: (1) in a pattern and (2) by the several pathophysiologic processes governed by IOP-related connective tissue stress and strain—regardless of the level of IOP at which these processes occur.

**Figure 1:**
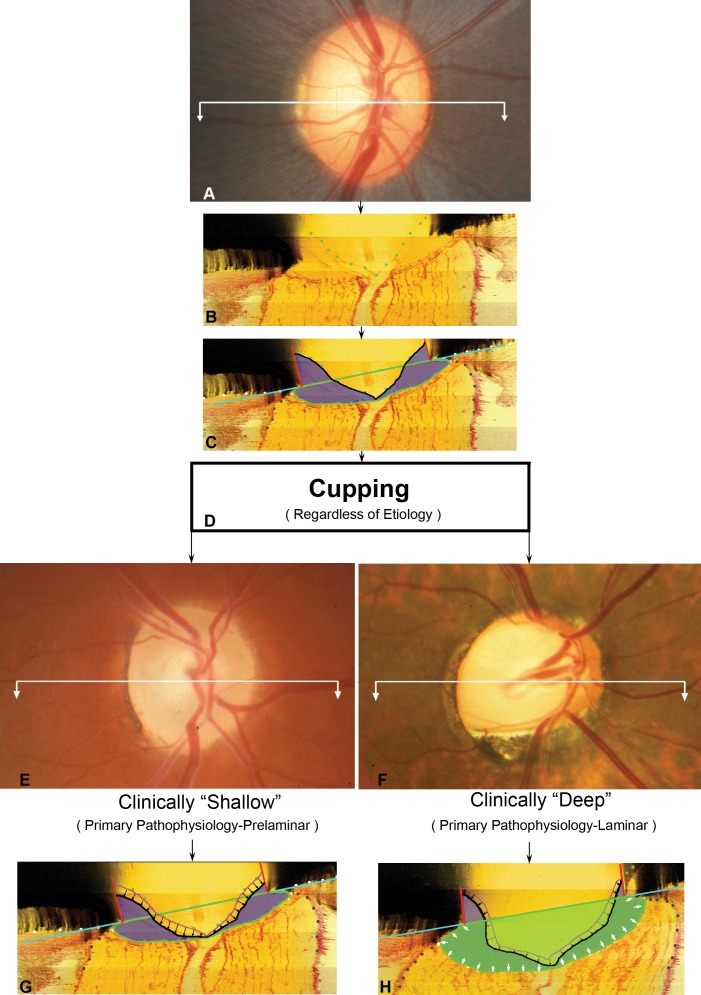
All clinical cupping, regardless of etiology, is a manifestation of underlying prelaminar and laminar pathophysiologic components. (**A**) Normal optic nerve head (ONH). To understand the two pathophysiologic components of clinical cupping, start with (**B**), a representative digital central horizontal section image from a postmortem 3D reconstruction of this same eye (*white section line* in [**A**]), vitreous top, orbital optic nerve bottom, and lamina cribrosa between the sclera and internal limiting membrane (ILM) delineated with *green dots*. (**C**) The same section is delineated into principal surfaces and volumes (*black*, ILM; *purple*, prelaminar neural and vascular tissue; *cyan-blue line*, Bruch's membrane opening [BMO] zero reference plane cut in section; *green outline*, post-BMO total prelaminar area or a measure of the space below BMO and the anterior laminar surface). (**D**) Regardless of the etiology, clinical cupping can be shallow (**E**) or deep (**F**) (these clinical photographs are representative and are not of the eye in [**A**]). A prelaminar or shallow form of cupping (**G**, *black arrows*) is primarily due to loss (thinning) of prelaminar neural tissues without important laminar or ONH connective tissue involvement. Laminar or deep cupping (**H**, *small white arrows* depict expansion of the *green-shaded space*) follows ONH connective tissue damage and deformation that manifests as expansion of the total area beneath BMO but above the lamina. Notice in (**H**) that while a laminar component of cupping predominates (*white arrows*), there is a prelaminar component as well (*black arrows*). Although prelaminar thinning is a manifestation of neural tissue damage alone, we propose that laminar deformation can occur only in the setting of ONH connective tissue deformation and remodeling. Reprinted with permission from Yang H, Downs JC, Bellezza A, et al. 3-D histomorphometry of the normal and early glaucomatous monkey optic nerve head: prelaminar neural tissues and cupping. *Invest Ophthalmol Vis Sci*. 2007;48:5068–5084.^3^

Regarding the character of these connective tissue alterations in glaucoma, we have identified five connective tissue components of ONH cupping in monkey unilateral experimental glaucoma: (1) posterior (outward) laminar deformation, (2) scleral canal expansion, (3) anterior (inward) and posterior migration of the anterior and posterior laminar insertions, (4) laminar thickness change (thickening in early deformation, thinning in advanced deformation), and (5) posterior bowing of the peripapillary sclera.^6^ We have also demonstrated early posterior deformation of the anterior lamina cribrosa surface (ALCS) that preceded the onset of detectable retinal nerve fiber layer thickness (RNFLT) thinning in a separate group of four young and four old monkeys with unilateral experimental glaucoma longitudinally followed with optical coherence tomography (OCT) imaging.^8^

In contrast to glaucoma, surgical optic nerve transection (ONT) is commonly used to study neuroprotection in the rat and mouse eye, as a model of non–IOP-related axonal degeneration.^9–11^ In the monkey eye, previous studies have described a lack of longitudinally detected surface deformation^12^ and prelaminar tissue thinning accompanied by pallor exceeding cupping that appears at approximately 6 weeks post transection.^11^ We recently performed longitudinal OCT imaging in five monkeys that underwent unilateral ONT surgery as part of a primary study of monkey ONH blood flow.^13^ Because the optic neuropathy of ONT is not thought to be “glaucomatous,” we hypothesized that the character of cupping within these longitudinal OCT data sets would be fundamentally different from our postmortem three-dimensional (3D) histomorphometric^6^ and longitudinal OCT^8^ characterizations of cupping in monkey experimental glaucoma. The purpose of the present study was to delineate and parameterize the longitudinal OCT data sets of these five ONT animals so as to test the hypothesis that ONH cupping in the monkey unilateral ONT model would include prelaminar and neuroretinal rim tissue thinning, but would not include posterior laminar deformation.

Why is it important to characterize the OCT phenotype of ONH cupping in the monkey ONT model? First, to demonstrate a longitudinal OCT (i.e., noninvasive) strategy for assessing the laminar and prelaminar components of cupping in the monkey eye that can be used in all forms of monkey^14^ and human^1,15^ optic neuropathy. Second, to further explore the hypothesis that posterior laminar deformation is a defining characteristic of a glaucomatous optic neuropathy, regardless of the level of IOP at which the neuropathy occurs or the primary mechanisms underlying it.^15–18^ Third, to establish support for the concept that RGC axon degeneration within the ONH that is secondary to an insult within the orbital optic nerve does not, in itself, drive the ONH cellular environment (astrocytes, microglia, oligodendrocytes, and scleral fibroblasts) into degenerative or remodeling pathways that leave the ONH and peripapillary scleral connective tissues vulnerable to OCT-detected deformation at normal levels of IOP (i.e., vulnerable to developing “normal-tension” glaucoma).

## Methods

### Animals

Five young adult (age 5.5–7.8 years) male rhesus monkeys (Macaca mulatta) were the subjects of a primary study on ONH blood flow changes in the unilateral ONT model.^13^ The present study tested hypotheses within the longitudinal OCT imaging that was performed as part of the original blood flow study. All procedures adhered to the ARVO Statement for the Use of Animals in Ophthalmic and Vision Research and were approved and monitored by the Institutional Animal Care and Use Committee at Legacy Research Institute (Portland, OR, USA). Table 1 provides a list of acronyms used in this report and their definitions.

**Table 1:**
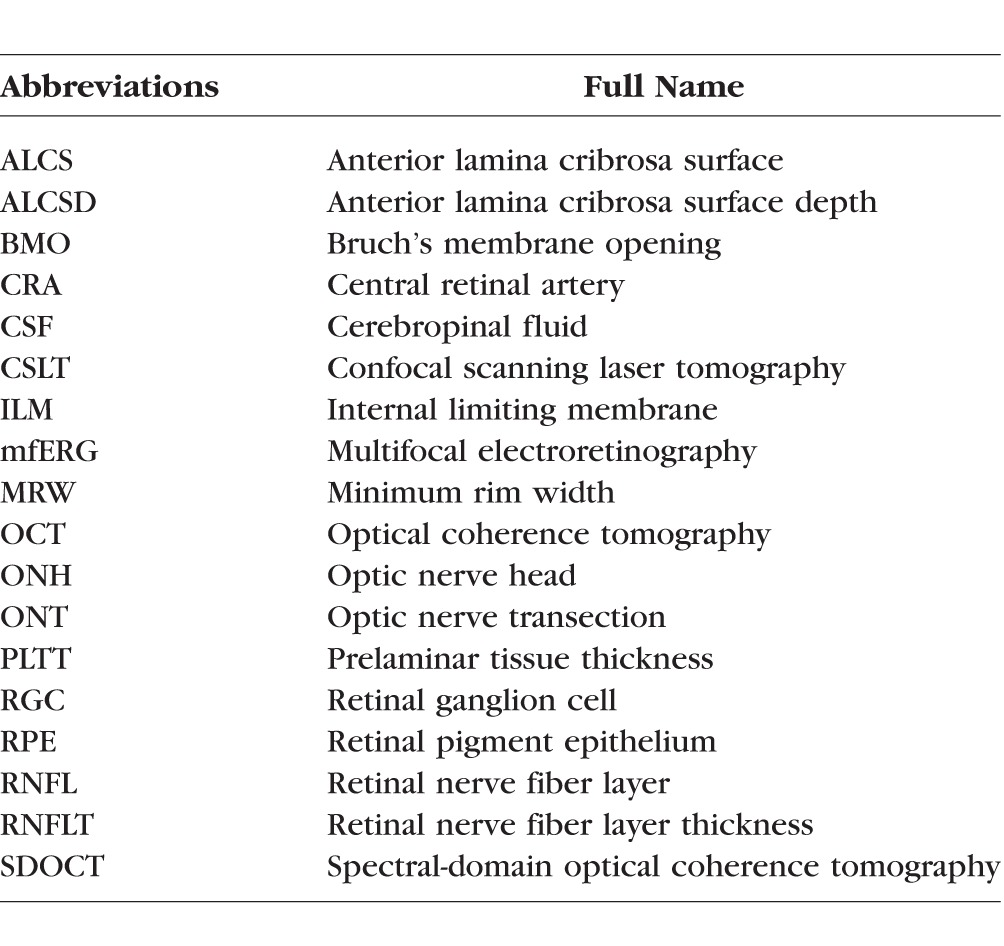
Abbreviations, Acronyms, and Definitions

### Overall Experimental Design

Both eyes of all animals underwent high-resolution (see below) OCT imaging (*n* = 5 times) for ONH and retinal nerve fiber layer (RNFL). Due to technical problems, high-resolution baseline data for the control eye in animal 5 was available only from the last two sessions (sessions 1–3 having been inadvertently acquired as low-resolution scans). Since staying within the same type of scan is required for proper disc and fovea–Bruch's membrane opening (BMO) alignment, we chose to drop the low-resolution scans and preserve precision between pre- and posttransection scans. Surgical ONT was completed in the right eye in each of the five monkeys (the ONT eye), while the left eye served as the control eye. Optical coherence tomography imaging was repeated in both eyes of each monkey every 6 to 12 days post ONT until *RNFLT* in the ONT eye was reduced by at least 40% of its baseline value (the endpoint of the primary blood flow study), at which time the animal was euthanized under deep anesthesia by perfusion fixation (details below). Please note that all measurement parameters are in italics to separate the OCT measurement of anatomic change (in italics) from the behavior of the anatomy itself (not in italics).

### Anesthesia

For all imaging, anesthesia was induced with intramuscular ketamine (15 mg/kg; Henry Schein Animal Health, Dublin, OH, USA) and xylazine (1.5 mg/kg; Akorn, Inc., Decatur, IL, USA), along with a single subcutaneous injection of atropine sulfate (0.05 mg/kg; Butler Schein Animal Health, Dublin, OH, USA). The animals were intubated and breathed air plus 10% oxygen spontaneously. Heart rate, end-tidal CO_2_, and arterial oxygenation saturation were monitored continuously. Body temperature was maintained at 37°C using a warming blanket. For the ONH blood flow and OCT imaging procedures, pupils were fully dilated with 1.0% tropicamide (Alcon Laboratories, Inc., Fort Worth, TX, USA). One of the superficial branches of a tibial artery was cannulated with a 27-gauge needle, which was connected to a pressure transducer (BLPR2; World Precision Instruments, Sarasota, FL, USA) and a four-channel amplifier system (Lab-Trax-4/24T; World Precision Instruments) for continuous arterial blood pressure recording throughout each experiment. Anesthesia was maintained by continuous administration of pentobarbital (8–12 mg/kg/h, intravenous) using an infusion pump (Aladdin; World Science Instruments, Inc., Sarasota, FL, USA). During the ONT surgical procedure, anesthesia was maintained by 1.5% to 3% isoflurane in oxygen.

### IOP Measurement and Control

Intraocular pressure was measured at each imaging session by handheld applanation tonometry (Tonopen XL; Reichert, Inc., Depew, NY, USA) in both eyes of each animal (a mean of 3 measurements per eye) within 30 minutes of general anesthesia induction.

### OCT and Fundus Imaging

**Figure 2:**
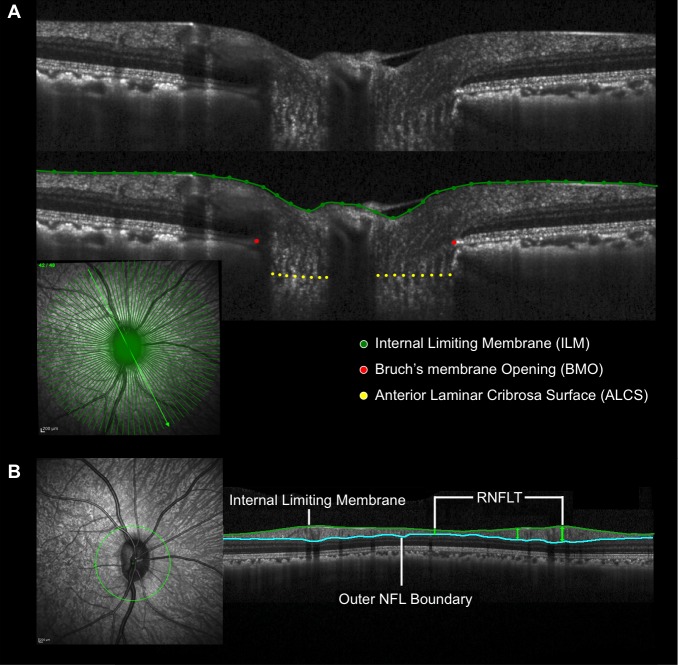
Original and delineated spectral-domain optical coherence tomography (SDOCT) optic nerve head (ONH) and retinal nerve fiber layer (RNFL) data sets in a normal monkey eye. (**A**) Forty-eight radial B-scans were manually delineated for each data set of each eye. *Green lines/points:* internal limiting membrane (ILM). *Red points*: Bruch's membrane opening (BMO). *Yellow points*: anterior lamina cribrosa surface (ALCS). (**B**) 12° circle scan of the ONH on the *left*. On the *right*, the 12° circle scan (approximately 3.0 mm from the ONH center), stretched out into a flat B-scan. Computer-delineated ILM and outer nerve fiber layer (NFL) boundary. Retinal nerve fiber layer thickness (RNFLT) measured by Spectralis software.

All imaging was performed 30 minutes after IOP was manometrically lowered to 10 mm Hg in both eyes. Forty-eight ONH radial B-scans (Fig. 1) and a 12° RNFL circle scan (Fig. 2) were obtained by spectral-domain OCT ([SDOCT], 1050-nm prototype Spectralis OCT; Heidelberg Engineering GmbH, Heidelberg, Germany). Radial B-scans were acquired over a 30° area (1536 A-scans per B-scan, *n* = 16 repetitions) followed by a 3.5-mm peripapillary RNFL circle scan (1536 A-scans per B-scan, *n* = 16 repetitions), which, when scaled for an average rhesus monkey axial length, is approximately a 3.00-mm peripapillary RNFL scan. All repetitive scans were acquired using eye tracking (Spectralis Manual) and averaged to reduce speckle noise. For each eye, the center of the ONH (as defined by the BMO) was estimated during the first imaging session and was used to align all follow-up images. Optic nerve head stereo photos and 30° color fundus photography were acquired using the Zeiss FF3 Camera (Carl Zeiss Meditech, Inc., Dublin CA, USA) during the first baseline and final imaging session.

### Optic Nerve Transection Surgery

Under general anesthesia (isoflurane, see above), a lateral orbitotomy was performed followed by a dissection of the lateral rectus muscle to expose the orbital optic nerve. Using limbal 6-0 Vicryl stay sutures the eye was rotated so that the central retinal artery (CRA) entrance into the sheath could be identified, the sheath above the entrance was cut open, and the nerve was severed behind the CRA approximately 6 to 8 mm behind the globe. While a full transection was attempted in each eye, where visibility made complete transection without CRA compromise uncertain, partial transections of at least two-thirds of the optic nerve diameter were accepted. Following transection, without closing the optic nerve sheath, the lateral rectus muscle and skin incisions were closed with sutures. Digital video fundus fluorescein angiography was performed 7 to 10 days after the procedure to verify the patency of central retinal vasculature in all eyes.^13^

### Animal Euthanasia and Retrobulbar Axon Counts

Animals were euthanized under deep anesthesia (intravenous SomnaSol, Henry Schein, Dublin, OH, USA) and tissues were preserved by perfusion fixation with 4% paraformaldehyde. A 2- to 3-mm sample of each optic nerve, beginning 2 mm posterior to the globe, was cut with a vibratome into 0.5-mm-thick transverse sections. Each section was postfixed in 4% osmium tetroxide and embedded in epoxy resin. Optic nerve cross sections (1 μm thick) were then cut and stained with p-phenylenediamine for axon counting. Axon counts for 100% of the optic nerve cross-sectional area were obtained by methods described in detail previously.^19^

### OCT Data Set Delineation and Parameterization

For each baseline and RNFL data set, the internal limiting membrane (ILM) and outer RNFL boundary within each standard 12° RNFL circle scan were segmented at the time of image acquisition using Spectralis automated software (HRA viewer v. 6.0.12.107; Heidelberg Engineering GmbH), then manually corrected by the technician as required and exported for *RNFLT* parameterization. For each baseline and post-ONT ONH data set, the ILM and BMO (innermost termination of the Bruch's membrane/retinal pigment epithelium [RPE] complex) within the 48 radial B-scans of each OCT ONH data were automatically segmented using Spectralis software (HRA viewer v 6.0.12.107), then exported into our in-house developed custom delineation software (ATL 3D) and corrected by a single delineator (EI). The same delineator then manually delineated the ALCS within the last three baselines and final imaging session ONH data sets only.

Within the ATL 3D delineating software, the ILM was delineated as discrete points interconnected by a Catmull-Rom spline; BMO was delineated using discrete points at either side of the neural canal opening; and the anterior lamina surface was delineated by placing discrete points where the feature was clearly visible (Fig. 2) based on our previous direct comparisons between OCT B-scans and matched histologic sections,^20^ as well as our previous publications on OCT laminar visualization^21^ and longitudinal change detection.^8^

**Figure 3:**
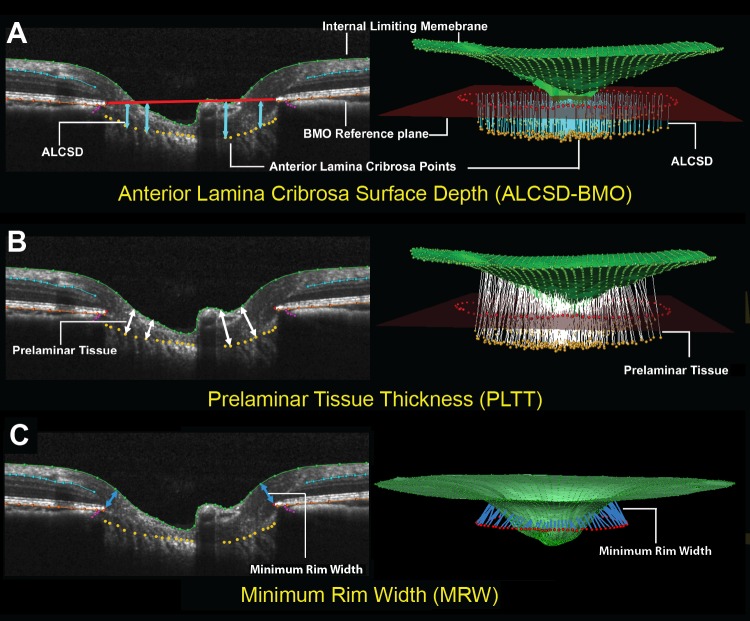
Three spectral-domain optical coherence tomography (SDOCT) optic nerve head (ONH) parameters. (**A**) Anterior lamina cribrosa surface (ALCS) depth (*ALCSD*) (*blue arrows*) is measured at each delineated ALCS point as the perpendicular distance from the Bruch's membrane opening (BMO) reference plane (*red line*). Note that by convention, *ALCSD* is a negative value when the ALCS is below the BMO reference plane. (**B**) Prelaminar tissue thickness (*PLTT*) is measured as the normal from the tangent to the ALCS to the internal limiting membrane (ILM, *green line*). (**C**) Minimum rim width (*MRW*, *blue arrows*) is measured at each delineated BMO point (*red*) as the minimum distance to ILM.

The parameter BMO–minimum rim width (*BMO-MRW* or *MRW*)^22^ (Fig. 3) was defined to be the minimum distance between the BMO and ILM calculated at each (*n* = 96) BMO point. Global *MRW* was defined to be the mean of the 96 individual MRW measurements. The parameter *RNFLT* was defined to be the minimum vertical thickness from the ILM to the outer RNFL boundary within each A-scan of the circle scan. Global *RNFLT* was defined to be the mean of all individual RNFLT measurements within the circle scan. A BMO reference plane was calculated based on the best-fitting ellipse through the 96 delineated BMO points as previously described.^23^ The parameter anterior lamina cribrosa surface depth (*ALCSD*) was calculated at each delineated ALCS point as the perpendicular distance to the BMO reference plane. Global *ALCSD* was defined to be the mean of all ALCSD measures within a given ONH data set. Post-ONT laminar deformation was defined as a change from baseline *ALCSD*. By our convention, *ALCSD* is negative when the lamina is below the BMO reference plane (Fig. 3). In the setting of anterior deformation of the ALCS, the parameter *ALCSD* becomes less negative (or more positive). In the setting of posterior deformation of the ALCS, the parameter *ALCSD* becomes more negative (or less positive). The parameter prelaminar tissue thickness (*PLTT*) was measured as the normal from the tangent of the ALCS to the ILM. Because the Spectralis x,y transverse pixel dimension is calibrated for human eyes, a scaling factor of 0.857 was employed to correct for ocular magnification differences in the monkey eye as previously described.^24^

### Data Analysis

The effect of ONT on IOP at baseline and post ONT was assessed by ANOVA using a linear mixed-effects model. The significance of overall (*n* = 5 animals) final imaging session change in each parameter was determined using a linear mixed-effects model (R Foundation for Statistical Computing, Vienna, Austria) in which the data from all five animals at baseline and final imaging were compared. To assess eye-specific change from baseline for each parameter, the baseline mean, SD, and 95% confidence interval (CI) were calculated for *RNFLT* and *MRW* in each eye using all five baseline testing sessions, and for *ALCSD* and *PLTT* using the last three baseline testing sessions. For each eye, the final imaging session value for each parameter was defined to be significantly different from its baseline value if the pre-euthanasia mean value fell outside of the baseline 95% CIs. Eye-specific change for the control eye of animal 5 could not be determined because 95% CI values could not be calculated from its *n* = 2 high-resolution baseline imaging sessions (see “Overall Experimental Design,” above).

## Results

### Animals

Five rhesus monkeys (aged 5.4–7.8 years) were included in this study (Table 2). Mean IOP at baseline was similar in pre-ONT versus control eyes (13.01 ± 1.07 and 13.46 ± 1.35 mm Hg, respectively; *t*-test: *P* = 0.6558). On average, post-ONT eyes demonstrated a slightly higher pressure compared to control eyes (mean ± SD: 12.84 ± 1.14 and 11.59 ± 0.83 mm Hg, respectively), though the difference was not significant (linear mixed-effects model, *P* = 0.2003). The percent total axon count reduction in the ONT eye (compared to its contralateral control eye) ranged from 47% to 72% (mean ± SD: 57 ± 12%, *P* = 0.004 paired *t*-test).

**Table 2:**
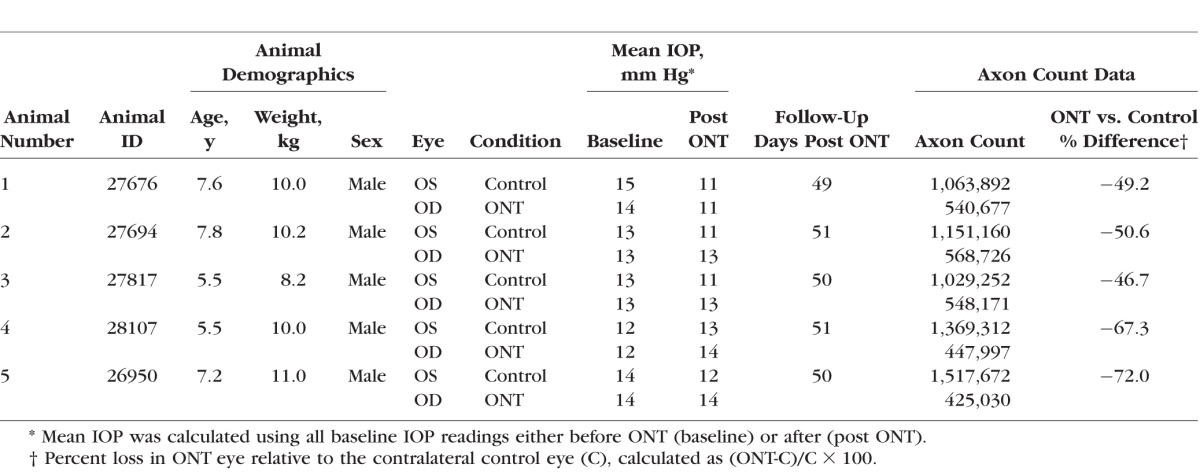
Demographic and IOP Parameters for Each Study Animal

### Experiment-Wide Final Imaging Session Change in Each Parameter

For all five animals considered together (Table 3), ONT eye change at the final imaging session was as follows: *MRW* (235.7 ± 22.3 μm) was significantly reduced from baseline (320.5 ± 38.4 μm) by −26.2% ± 3.3% (*P* = 0.0011); *RNFLT* (61.3 ± 4.5 μm) was significantly reduced from baseline (109.2 ± 6.8 μm) by −43.8% ± 3.7% (*P* < 0.0001); *PLTT* (358.3 ± 80.3 μm) was significantly reduced from baseline (468.8 ± 91.5 μm) by −23.8% ± 3.2% (*P* = 0.0013); and *ALCSD* (−167.1 ± 36.5 μm) was significantly anteriorly displaced from baseline (−211.4 ± 21.5 μm) by 20.8% ± 15.2% (*P* = 0.033). Within the control eyes, *PLTT* and *MRW* were significantly increased at the final imaging session (38.9 ± 22.7 μm [*P* = 0.0217] and 20.0 ± 15.1 μm [*P* = 0.032]), respectively, compared to baseline (see Table 3).

**Table 3:**
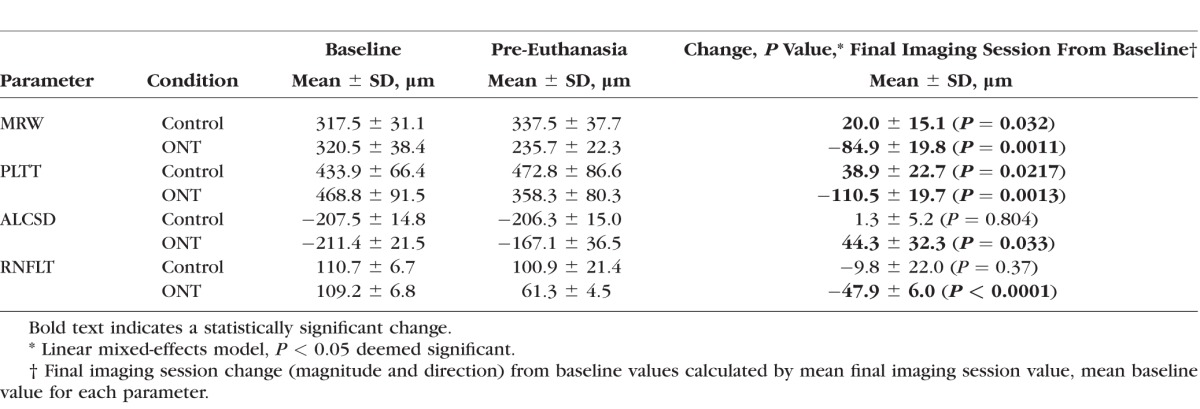
Experiment-Wide Baseline, Final Imaging Session, and Change Values for Each Parameter Within Control and ONT Eyes

### Eye-Specific Final Imaging Session Change in Each Parameter

**Figure 4:**
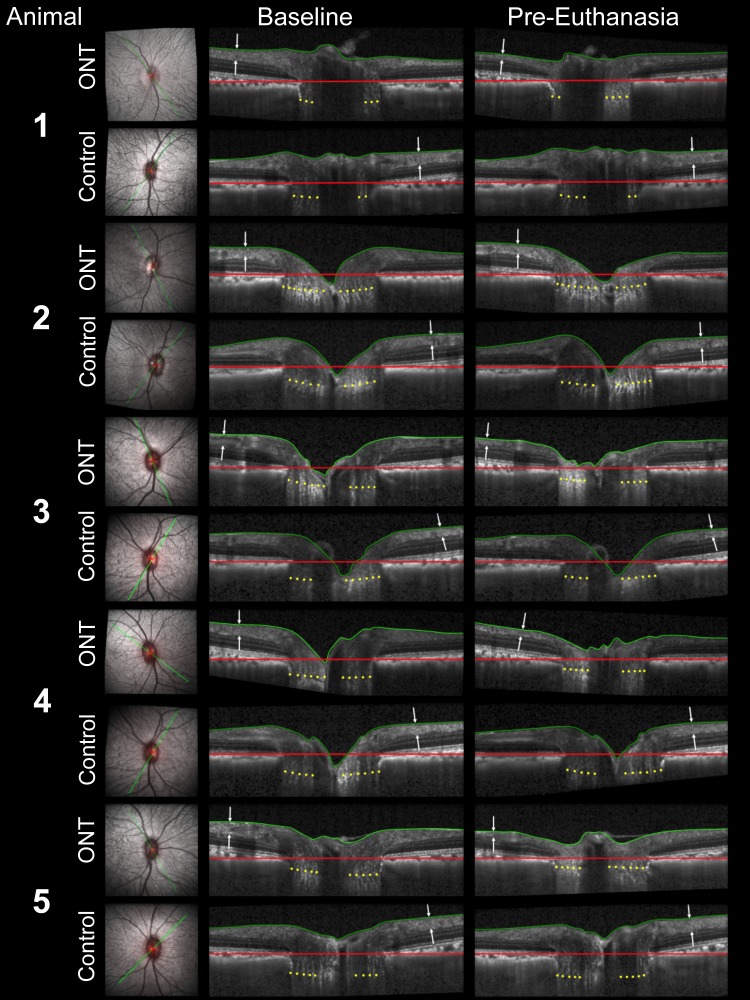
Representative baseline (*left*) and pre-euthanasia (*right*) radial B-scans from the optic nerve transection (ONT) and control eyes of all five study monkeys. Scanning laser ophthalmoscopy image (*left*) showing radial B-scan location in each eye (*green line*). Within each baseline and pre-euthanasia radial B-scan the following landmarks are delineated: internal limiting membrane (ILM, *green line*); Bruch's membrane opening (BMO) reference plane (*red line*); and the anterior lamina cribrosa surface (ALCS, *yellow dots*). ONT eye retinal nerve fiber layer thickness (RNFLT, *white arrows*) is markedly thinned within the pre-euthanasia compared to the baseline B-scans, while control eye RNFLT remains unchanged in all animals. ONT eye ALCS position relative to the BMO reference plane remains unchanged in M1 and M2 (and moves anteriorly in M3, M4, and M5), while laminar position remains unchanged in all control eyes.

The frequency, magnitude, and direction of eye-specific final imaging session change within the five ONT (animals 1–5) and four control (animals 1–4) eyes in which it could be assessed (see Methods) are summarized in Table 4. All five ONT eyes demonstrated significant decreases in *MRW* (range, −62.4 to −116.1 μm [−23.7% to −31.8%]), *RNFLT* (range, −39.9 to −51.2 μm [−39.6% to −49.5%]), and *PLTT* (range, −85.7 to −130.2 μm [−23.0% to −28.2%]). Anterior lamina cribrosa surface depth was unchanged in two ONT eyes (animals 1 and 2), and was significantly anteriorly displaced in animals 3, 4, and 5 (range, 55.8 to 80.8 μm [25.7% to 39.2%], *P* = 0.033). No control eye change was detected for *RNFLT* and *ALCSD*. However, *PLTT* was increased in three of the four control eyes in which it could be assessed (animals 1, 3, and 4; range, 42.1 to 69.2 μm [9.8% to 13.5%]), and *MRW* was increased in one control eye (animal 2; 40.0 μm [13.0%]). Representative baseline and final imaging session B-scan images of the control and ONT eye of all five animals are shown in Figure 4.

**Table 4:**
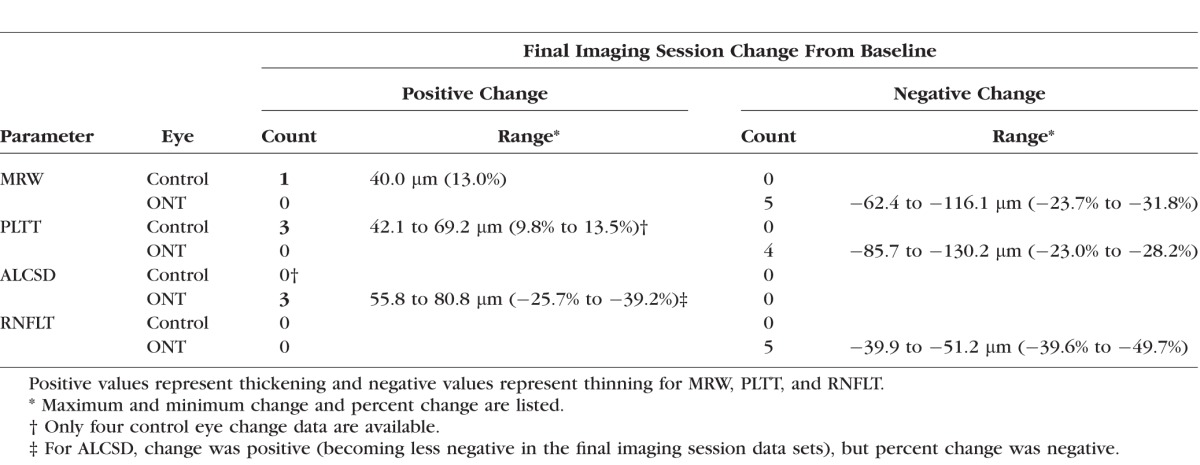
Frequency, Magnitude, and Direction of Control and ONT Eye-Specific Change for Each Parameter

### Longitudinal Change in MRW and RNFLT

**Figure 5:**
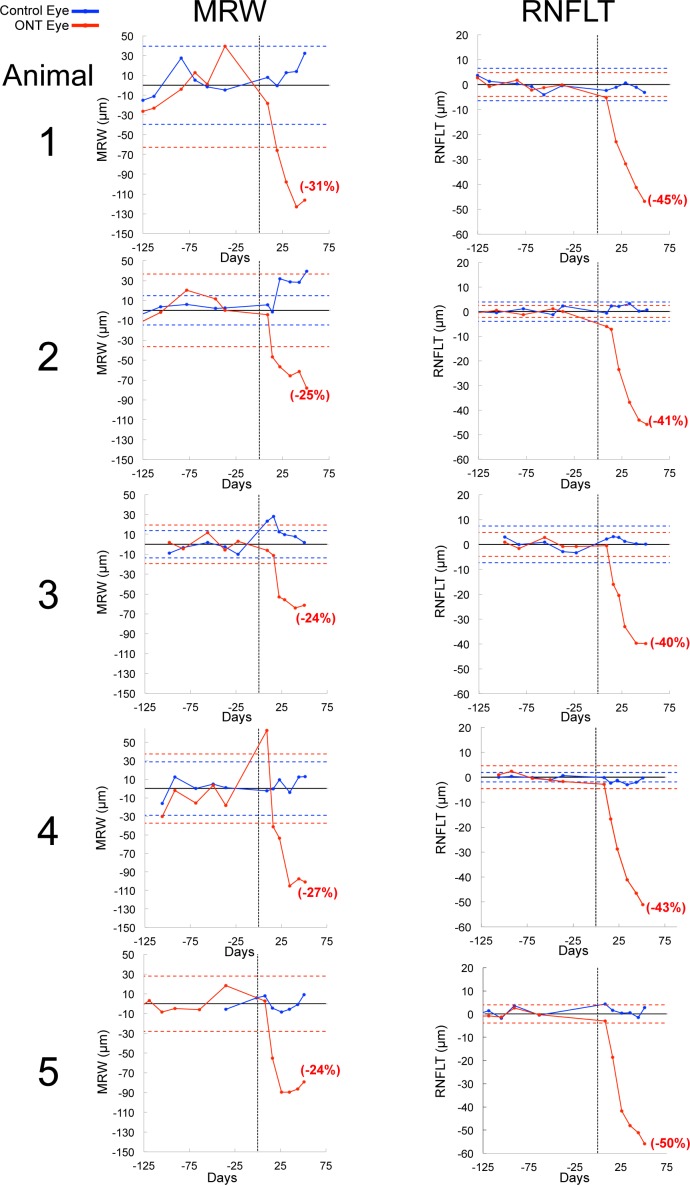
Spectral-domain optical coherence tomography (SDOCT) global minimum rim width (*MRW*) and retinal nerve fiber layer thickness (*RNFLT*) pre- and post-optic nerve transection (ONT) change from baseline mean. Data for both the ONT and control eyes are normalized to the baseline mean for that eye by subtracting the baseline mean value at each imaging time point. For each animal, the following data are displayed: *vertical dashed black line*, day 0 = date of ONT; *horizontal black line*, for each eye zero change from its baseline mean value; *horizontal dashed blue lines*, the 95% confidence interval for the control eye based on the baseline sessions; *horizontal dashed red lines*, the 95% confidence interval for the (future) ONT eyes based on the baseline sessions. The percent changes (calculated from the mean of the baseline time points) for *RNFLT* and *MRW* are listed in *red parentheses* for each ONT eye at the final imaging sessions. By convention, negative values for *RNFLT* and *MRW* indicate thinning. Positive values for *RNFLT* and *MRW* indicate thickening. Note that the control eye of animal 2 demonstrates an increase in *MRW* that exceeds its baseline 95% confidence interval for that parameter. Note also that a 95% confidence interval could not be generated for the control eye of animal 5 (see Methods).

**Figure 6:**
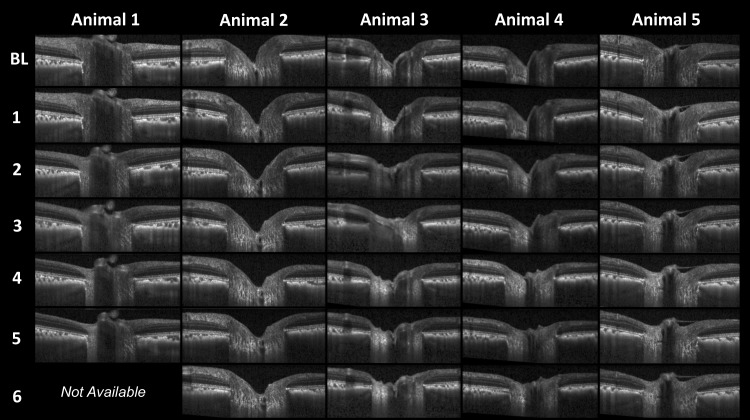
Representative B-scans from all post-optic nerve transection (ONT) imaging sessions for each ONT eye. *Top row* represents baseline (BL) scan and *rows below* are imaging sessions post ONT (each session 6–11 days apart). Note the significant anterior movement of the lamina cribrosa, flattening of the optic cup, and thinning of the retinal nerve fiber layer over time. The B-scans for each eye are in locations similar to those shown in Figure 4 so as to avoid the principal retinal vessels.

Plots showing change in MRW and RNFLT at each post-ONT imaging session relative to their baseline 95% CIs are shown in Figure 5. Representative OCT B-scans from each postlaser imaging session are shown for each ONT eye in Figure 6. These two figures together demonstrate that while substantial thinning of the rim and RNFL occurred in all five ONT eyes, posterior laminar deformation did not occur in a single ONT eye.

## Discussion

What constitutes a glaucomatous form of cupping remains undefined in the human,^1,15,25^ monkey,^14,26^ tree shrew,^27^ rat,^28,29^ and mouse^30,31^ eye. Other aspects of the clinical behavior of the neuropathy are often helpful in making this determination, such as the pattern of loss within the neuroretinal rim tissue, peripapillary RNFL tissue, and visual field, as well as the assessment of optic disc pallor. However, neither a quantitative description of glaucomatous structural change nor a strategy for its staging (independent of the magnitude of RGC axon loss)^6^ currently exists. Building the tools for characterizing the phenotype of a glaucomatous optic neuropathy is necessary for human patient care^1,15^ (i.e., the detection of structural glaucoma) and also necessary for the development of animal models of glaucoma that occur at normal^26,32,33^ and elevated levels^6,14,34,35^ of IOP.

We recently reported the character of connective tissue deformation and remodeling in early through end-stage monkey experimental glaucoma using postmortem, 3D histomorphometry,^6^ in which profound laminar deformation and remodeling were present in eyes with as little as 12% to 16% postmortem optic nerve axon loss. In a separate group of animals, we longitudinally characterized ONH and RNFL change at the onset of early monkey experimental glaucoma using OCT.^8^ In that study,^8^ early posterior laminar deformation was detectable in eyes with no detectible RNFL change and no detectible axon loss once the animal had been euthanized.

**Figure 7:**
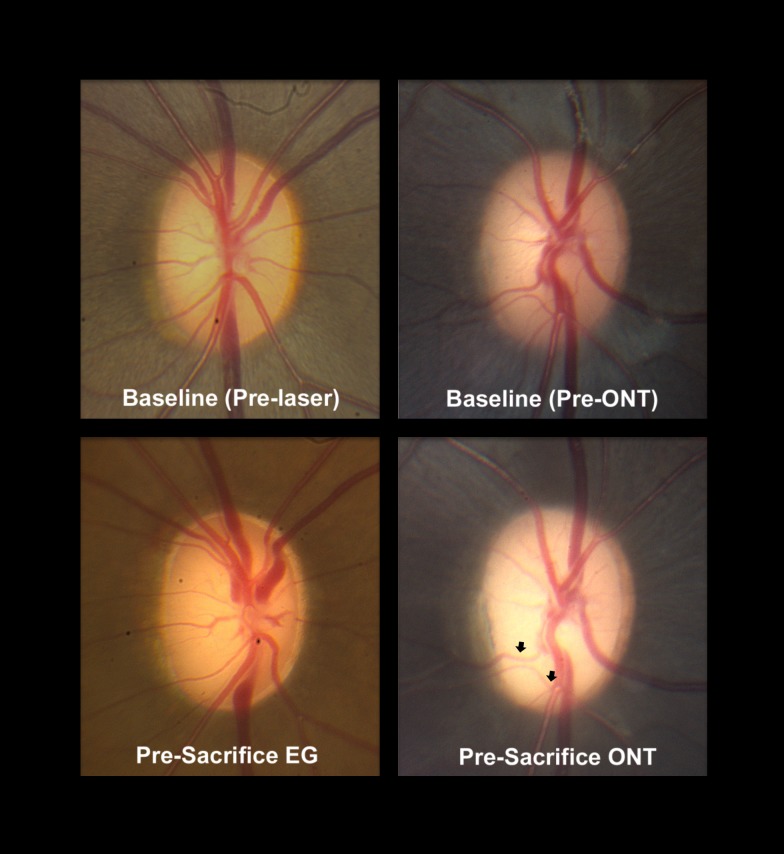
Cupping in a representative monkey experimental glaucoma (EG, *left*) and optic nerve transection (ONT) eye (animal 2, *right*). *Left*: representative EG eye (monkey 13904, not in this study) at baseline (prior to laser, *above*) and near the time of euthanasia (*below*) from an old (16.1 years of age) animal with 58% axon loss. *Right*: representative young adult ONT eye (animal 2, from this study) (7.8 years old) with 51% axon loss. Both eyes are shown in right eye orientation. In the EG eye (*left images*), note the posterior deformation and early excavation of the central retinal artery and veins as they leave the lamina and cross the clinical disc margin (which in this eye is Bruch's membrane opening [BMO] by optical coherence tomography [OCT], not shown). Early “nasalization” of the vessels and “bayoneting” of the inferior vein as well as diffuse loss of the retinal nerve fiber layer (RNFL) striations are also apparent. In the ONT eye (*right images*) while diffuse pallor and RNFL loss (−41% by OCT) are apparent, even in the face of OCT-detected prelaminar and rim tissue thinning, the presence of clinical cupping is not apparent, being suggested only by a slight change in the trajectory of the inferior temporal vessels (*black arrows*). No eye-specific change in anterior lamina cribrosa surface depth was detected by OCT in this eye (see Results).

In the present study, we asked the questions: Do the tissues of the monkey ONH demonstrate cupping in a non–IOP-related form of RGC axon injury induced by surgical transection of the optic nerve, and if they do, is there a “laminar” or “connective tissue” component (Fig. 1)? Both our overall and eye-specific analyses establish that rim thinning and prelaminar tissue thinning were present at levels of RNFL thinning ranging from −39.6% to −49.5% and levels of optic nerve axon loss ranging from −46.7% to −72%. This finding agrees with a previous histologic report^11^ that described prelaminar tissue thinning reaching a maximum of 50% at 8 weeks post ONT in the monkey eye. Not surprisingly, similar to observations in the same previous report,^11^ ONH pallor exceeded cupping in each of the ONT eyes as shown for animal 2 in Figure 7. Regarding the issue of posterior laminar deformation, not only did this not occur in a single ONT eye, but anterior (inward) laminar deformation was detected in three of the five ONT eyes. While our findings are not unexpected, they clearly establish that the shallow cupping that is present in the optic neuropathy of ONT does not include a “laminar component.”

Considering the importance of phenotyping the connective tissue components of a glaucomatous optic neuropathy, we recently reviewed this subject as it pertained to all existing experimental and spontaneous optic neuropathies in the monkey eye,^14^ and proposed landmarks and parameters regionalized relative to the axis between the fovea and BMO centroid for this purpose.^14,36^ Regarding the evidence for laminar deformation in the existing monkey optic neuropathies, an idiopathic bilateral optic neuropathy that demonstrated nonglaucomatous pallor of the ONH, accompanied by RNFL thinning most predominant within the maculopapular bundle, has been described in nine monkeys of Chinese origin obtained from two different primate centers.^37^ However, no in vivo or postmortem histologic assessment of laminar anatomy was performed. Monkey models for unilateral anterior ischemic optic neuropathy,^38,39^ ONT,^9–12,40,41^ and chronic optic nerve endothelin exposure,^42–45^ as well as bilateral optic neuropathy following primary cerebrospinal fluid (CSF) lowering,^26^ have been described. Of these, the neuropathies in the anterior ischemic optic neuropathy and ONT models demonstrate mild (transection) to profound (anterior ischemic) disc swelling followed by diffuse pallor without evident cupping. No direct assessment of connective tissue deformation has been made.

In the implanted endothelin pump model of unilateral optic nerve vasoconstriction, after preliminary studies in rabbits,^46,47^ optic nerve blood flow reduction in monkeys was characterized,^45^ and localized optic nerve axon loss in the setting of diffuse RNFL loss with shallow cupping was reported in a subset of 12 monkeys.^44^ Chauhan et al.^48^ then reported optic nerve axon and RGC loss but infrequent (1 of 21 eyes) ONH topographic change in the rat endothelin optic neuropathy model. A follow-up monkey study designed to detect longitudinal confocal scanning laser tomography (CSLT) and scanning laser polarimetry change, as well as postmortem laminar deformation within the endothelin-treated eyes of a separate group of animals, failed to achieve detectable optic nerve axon loss in the endothelin eyes and its results were therefore uninterpretable (Jack Cioffi, oral communication, April 2015). In a later study in five rhesus macaques unilaterally implanted with endothelin pumps and followed for 1.5 years,^42^ no significant changes in ONH morphology or ONH blood flow velocity were detected by CSLT and laser Doppler flowmetry, respectively. In that study, optic nerve axon counts were also not significantly decreased in the endothelin-treated eyes.

Yang et al.^26^ reported diffuse RNFL and ONH rim thinning in two of four monkeys following surgical CSF pressure lowering. A third monkey demonstrated a single nerve fiber hemorrhage, but no other change. While quantitative assessment was not reported, no qualitative evidence of laminar deformation was present within the published OCT images. Subsequent unpublished quantification has confirmed that there was no OCT-detected laminar deformation within the four studied animals (Ningli Wang, oral communication, April 2015). While the appearance of this neuropathy is not glaucomatous by the criteria suggested above, the model is important because it demonstrates that primary CSF lowering at “normal” levels of IOP is a risk factor for RGC axon loss in a subset of monkey eyes. It therefore also suggests that in a given eye, a relative increase in the translaminar pressure gradient (by whatever cause) may be a risk factor for RGC axon loss at all levels of IOP.

Regarding the importance of detecting connective tissue deformation and/or remodeling in experimental models of human normal-tension glaucoma, why should we expect the lamina to deform posteriorly when IOP is “normal”? While there is literature suggesting that the clinical phenotypes of glaucoma that occur at normal and elevated levels of IOP are different,^49–52^ we propose that glaucoma represents a continuum of ONH susceptibility to IOP, IOP-related, and non–IOP-related risk factors. Within this continuum, IOP should be a substantial risk factor at normal levels of IOP,^53^ its contribution to risk should increase as it becomes elevated,^54^ and the presence of IOP-related and non–IOP-related risk factors at any level of IOP should increase the risk of IOP, alone.^53,55^ While there is substantial diversity in the appearance of cupping in all forms of glaucoma^56^ that includes a shallow, “senile sclerotic” form (most common in elderly eyes),^56,57^ the simple clinical truth is that the majority of normal-tension glaucoma eyes “cup” in the absence of detected IOP elevation, in a manner indistinguishable from that for eyes with chronic IOP elevation.^58^ Several recent cross-sectional studies have used OCT imaging to detect laminar deformation^16,59^ in human normal-tension glaucoma eyes. While prospective, longitudinal OCT-based evidence is necessary, it is reasonable to propose that the connective tissues of normal-tension glaucoma eyes deform and remodel under the level of engineering stress and strain that are generated by statistically normal levels of detected IOP and statistically normal translaminar pressure gradients. Their connective tissue susceptibility may be the result of their innate (nonperturbed) condition, or may follow from a separate, non–IOP-related, primary insult^32,33^ that leaves them susceptible to a previously tolerated normal level of IOP.

It is also possible that the non–IOP-related components of susceptibility such as ischemia, autoimmunity, inflammation, and low CSF pressure, which would be present at all levels of IOP, are more prominent in those eyes that develop the neuropathy at normal IOP levels. If this is true, their presence may influence the character of the neuropathy in ways that are separate from the shallower form of cupping we would expect from the fact that normal-tension glaucoma occurs most commonly in aged eyes (which are stiff and/or senescent), and at lower levels of IOP (i.e., at lower translaminar pressure gradients), which both lessen the likelihood of deep deformation and extensive connective tissue remodeling.

With regard to the anterior laminar displacement that occurred in three ONT eyes in this study, several phenomena may have contributed to this finding. First, IOP may have been lower in the ONT eyes post transection. However, detected IOP was in fact higher in the ONT eyes, though the difference did not achieve significance. Second, retrolaminar tissue pressure may have been higher in the ONT eyes post transection. However, with an open subarachnoid space following transection, CSF would most likely be lower rather than higher, and retrolaminar tissue pressure would most likely follow CSF pressure, as shown in previous studies by Morgan and coauthors.^60,61^ Third, anterior scleral canal expansion has previously been shown to pull the lamina anteriorly within the scleral canal.^62^ Thus, with the dramatic anterior laminar displacement seen by us, it might be possible that scleral canal expansion could follow this change. It is also possible that primary RGC axon degeneration caused a change in scleral material properties that led to scleral canal expansion (without IOP elevation). However, our SDOCT data failed to detect anterior scleral canal expansion in the one ONT eye in which the anterior scleral canal opening could be measured (animal 5, data not shown). Fourth, astrocyte reorganization into the laminar pores in response to axon loss has previously been described in monkey ONT models.^9,11^ Contraction of this tissue combined with residual edema may have contributed to anterior laminar surface displacement.

Finally, the fact that we detected final imaging session control eye increases in *MRW* and *PLTT*, both overall and in one (*MRW*) and three (*PLTT*) of the four control eyes in which eye-specific change could be assessed, warrants further discussion. First, this swelling may be related to the placement of the anterior chamber needle and or manometer fluid that was used in both eyes of each animal to control IOP at 10 mm Hg during each OCT imaging session. Or it may be the cumulative result of prolonged hypotony following needle removal after each imaging session that, by the end of post-ONT imaging, resulted in a detectable level of ONH neural tissue swelling. However, in a previous longitudinal OCT imaging study in eight monkeys with unilateral experimental glaucoma in which manometer-controlled IOP 10 mm Hg imaging was also performed,^8^ a pre-euthanasia control eye decrease in *MRW* was detected in one animal by event analysis. A control eye increase in *PLTT* was detected in a second animal by trend analysis.

Several pieces of evidence suggest there may be permanent control eye alterations in monkey experimental glaucoma and ONT. First, transynaptic degeneration of RGCs has been demonstrated in the monkey eye following primary unilateral removal of the visual cortex.^63^ Second, while multifocal electroretinography (mfERG) testing was not performed in the present study, the Fortune laboratory has reported control eye longitudinal loss of mfERG signal in pooled data from a total of 39 unilateral monkey experimental glaucoma animals.^64^ Because of these concerns, a careful comparison of control eye orbital optic nerve axon number, size, and shape compared to “naive” normal eyes from bilateral-normal monkeys will be undertaken in the control eyes of both ONT and experimental glaucoma animals. The presence of control eye optic nerve axon loss or degeneration, if detected, will be separately reported and its mechanisms then pursued.

Our study is limited by the following considerations. Animals were young adults and our follow-up, being dictated by the primary study,^13^ extended through 40% *RNFLT* loss (49–51 days post ONT). This may not have been enough time for laminar deformation to develop, and/or in young adults the connective tissue response to primary insult may be different than in old eyes. It is possible that in old eyes, connective tissue and extracellular matrix remodeling may have preferentially degraded and weakened the ONH connective tissues, making them susceptible to deformation at normal levels of IOP. Longer studies in older animals are necessary to rule out posterior laminar deformation as a late development in this neuropathy.

Finally, the ONTs we performed were likely incomplete in four of the five animals by clinical inspection, which suggested superior nasal or nasal sparing in four of the five ONT eyes. However, for the purposes of testing hypotheses regarding laminar deformation, *RNFLT* in all animals was above 40% and optic nerve axon loss ranged from 47% to 72%. While complete transections, followed for several years, would give us the most definitive answers to the questions we posed, cupping by all measures including posterior laminar deformation is profound within experimental glaucoma eyes at similar levels of *RNFLT* and axon loss as in the ONT animals of this study (Fig. 7).

In summary, our study characterizes ONH cupping in the monkey ONT optic neuropathy model and finds prelaminar and rim tissue thinning without evidence for posterior laminar deformation. We propose that the strategies we have described are applicable to all forms of optic neuropathy in the monkey^14^ and human eye^15^ and can be used to longitudinally address the question of phenotype in future models of monkey experimental glaucoma that do not include IOP elevation.
